# Use of immunology in news and YouTube videos in the context of COVID-19: politicisation and information bubbles

**DOI:** 10.3389/fpubh.2024.1327704

**Published:** 2024-02-07

**Authors:** Rachel Surrage George, Hannah Goodey, Maria Antonietta Russo, Rovena Tula, Pietro Ghezzi

**Affiliations:** ^1^Brighton and Sussex Medical School, Brighton, United Kingdom; ^2^Department of Biomolecular Sciences, University of Urbino, Urbino, Italy

**Keywords:** COVID-19, YouTube, news, science communication, scientific journalism, health information

## Abstract

**Background:**

The COVID-19 pandemic propelled immunology into global news and social media, resulting in the potential for misinterpreting and misusing complex scientific concepts.

**Objective:**

To study the extent to which immunology is discussed in news articles and YouTube videos in English and Italian, and if related scientific concepts are used to support specific political or ideological narratives in the context of COVID-19.

**Methods:**

In English and Italian we searched the period 11/09/2019 to 11/09/2022 on YouTube, using the software Mozdeh, for videos mentioning COVID-19 and one of nine immunological concepts: antibody-dependent enhancement, anergy, cytokine storm, herd immunity, hygiene hypothesis, immunity debt, original antigenic sin, oxidative stress and viral interference. We repeated this using MediaCloud for news articles.

Four samples of 200 articles/videos were obtained from the randomised data gathered and analysed for mentions of concepts, stance on vaccines, masks, lockdown, social distancing, and political signifiers.

**Results:**

Vaccine-negative information was higher in videos than news (8-fold in English, 6-fold in Italian) and higher in Italian than English (4-fold in news, 3-fold in videos). We also observed the existence of information bubbles, where a negative stance towards one intervention was associated with a negative stance to other linked ideas. Some immunological concepts (immunity debt, viral interference, anergy and original antigenic sin) were associated with anti-vaccine or anti-NPI (non-pharmacological intervention) views. Videos in English mentioned politics more frequently than those in Italian and, in all media and languages, politics was more frequently mentioned in anti-guidelines and anti-vaccine media by a factor of 3 in video and of 3–5 in news.

**Conclusion:**

There is evidence that some immunological concepts are used to provide credibility to specific narratives and ideological views. The existence of information bubbles supports the concept of the “rabbit hole” effect, where interest in unconventional views/media leads to ever more extreme algorithmic recommendations.

## Introduction

The COVID-19 pandemic engendered a significant amount of new media coverage. A study of online news sources reported that, in the period between January and October 2020, the pandemic represented roughly 25% of new articles in most countries including Italy, the United Kingdom and the United States (1). While communicating health-related matters is always challenging, it was particularly difficult to articulate the uncertainty around COVID-19 prevention and treatment, as well as the often changing guidelines from the WHO and local health authorities (2–5). This proved fertile ground for misinformation and disinformation, including the development of conspiracy theories, and many studies have addressed this so-called “infodemic,” analysing news, video content and social media (6).

Anxiety in general (7) and, more specifically, inability to cope with uncertainty, increases susceptibility to conspiratorial thinking and misinformation (8, 9). This may have consequences, as harbouring conspiratorial beliefs decreases adherence to health authority guidelines, including the uptake of vaccines (10, 11).

Most governments claimed that they “followed the science” when making public health decisions (12–14), and as such concepts like “herd immunity,” (13) often explained by experts (15) in the mass media, responded to the public “need for orientation”(16). The quality of scientific communication varied greatly: one study of German news reported that experts used by the media had higher reputations and expertise in the field (15), while others found a disconnect between the presence of experts in the news in multiple countries, including the United States, and their track record of publication (17); some even noting a “convergence of science journalism and self-communication of scientists” (18).

A well-studied characteristic of the media environment during the pandemic is the high degree of politicisation of various non-pharmacological measures adopted, such as lockdowns, social distancing and face masks, as well as of pharmacological interventions, both preventative (vaccines) and potentially therapeutic (ivermectin and hydroxychloroquine, in particular). This was associated with the spread of misinformation, even around vaccines that had undergone clinical trials according to standard evidenced-based procedures, that may have resulted in a decrease in vaccination rate (19, 20). The politicisation of the COVID-19 pandemic affected the mass media and Leidecker-Sandmann reported that, in German news, political actors were mentioned more frequently than scientists, something that was not observed in previous pandemics (15). Science was itself not exempt from politicisation either, with Bozeman arguing that “hyper-politicization of science” affected use (and misuse) of scientific and technical information (12).

Polarisation of scientific issues in the media, such as in the case of biological evolution, can influence the choice of expert by news media (21). Journalists can use experts not only to help interpret or explain difficult scientific concepts but also to support their pre-constructed narrative; in the latter case experts are used to provide what Albaek defined “compensatory legitimation” (22) to increase the credibility of their content.

Likewise, scientific concepts could be twisted, mis-interpreted and “used” to support a particular view of a public health issue, such as the COVID-19 pandemic. The aim of this project was to study the use of nine scientific concepts, listed in Table 1, related to immunology or immunopathogenesis that were discussed in the media in the context of the COVID-19 pandemic.

**Table 1:** Immunological concepts investigated.

Cytokine storm. Well-established concept with over 4,000 hits in PubMed on COVID-19. Activation of innate immunity leads to overproduction of inflammatory cytokines (IL-1, 6, TNF etc) that cause hyperinflammation and tissue damage. This concept is at the basis of the use of anti-inflammatory agents (anti-IL-6, dexamethasone) (23, 24).
Oxidative stress. Well-established theory. Infection and inflammation can cause overproduction of oxidants (reactive oxygen species) that would then cause tissue damage. PubMed shows 866 studies on oxidative stress and COVID in 2021–2022 but no antioxidant has been approved so far for any indication (25, 26).
Immunity debt. New concept, not mentioned in immunology textbooks. Lack of exposure to pathogen due to NPIs would lead to lack of immunity against them. PubMed shows just 11 papers, all of them in 2021–2022 and on COVID, all mentioning immunity debt as a hypothesis. This concept is often confused with the hygiene hypothesis (below) (27).
Hygiene hypothesis. Well-established theory but only 16 hits in PubMed on it and COVID-19. States that hygiene causes lack of exposure to parasites, dampening Th2 responses and thus causing increased allergic disease (asthma, hay fever and other allergic disease) or autoimmune disease (multiple sclerosis) (28, 29).
Herd immunity. Well-established concept in vaccinology by which when a given percentage of a population is immunised, the whole community is protected from the spread of an infection (30).
Viral interference. Well-established concept since the discovery of “interferons” in 1957. Viral infection causes induction of interferons, antiviral cytokines that inhibit the replication of many viruses. PubMed shows 33 papers on this and COVID-19 with some research studies. Should note, however, that viral interference is not lasting “immunity” as interferon induction is short-term (31).
Antibody-dependent enhancement (ADE). For some viruses (Dengue, in particular), binding to an antibody (either infection-or vaccine-induced) may increase their entry into the cell (exploiting the Fc receptors). There are studies suggesting ADE for COVID-19 but the well-demonstrated efficacy of monoclonal antibodies and vaccines rules out the possibility that they may worsen infections via ADE (32).
Anergy, t-cell exhaustion, immunoparalysis. Some of these are established concepts. Overstimulation of T cells with an antigen in chronic infections results in their inhibition (anergy). This is a mechanism that prevents immune responses against self-antigens (tolerance). Despite the use of the concept by anti-vaxxers, this has never been observed with vaccines. Only 4 papers in PubMed on lymphocyte anergy and COVID-19 exist, one showing the opposite (infection dampens anergy) (33, 34).
Original antigenic sin, immune/antigenic imprinting. The immune system responds with immunological memory based on a previous infection when encountering a variant of the same virus. Evolved to promote a rapid antibody response against conserved antigens. PubMed shows 30 studies on COVID-19, some research studies (35–37).

To investigate whether these were used to support specific narratives around vaccines and non-pharmaceutical interventions, we studied whether their mention correlated with a negative stance towards these interventions and if they were associated with politicisation of the discourse.

The study used a sample of US and Italian news articles obtained from the database MediaCloud (*), and YouTube videos in English or Italian. The results suggest that while some concepts were “neutral,” others were more present in “information bubbles,” often with a high degree of politicisation.

## Methods

### Database search

Search queries are described in the supplementary material (Supplementary Table S1) and were limited to the period 11/09/2019 and 11/09/2022. News articles were searched and downloaded from MediaCloud^1^ using the geographical collections “United States – National,” “Italy-National” and “Italy – State & Local.” The results were downloaded to a spreadsheet along with their metadata (e.g., date, URL, title, media name). Then, their order was randomised using the RAND function in Excel and 25 results (or fewer, when those were < 25) for each search term were combined into a single spreadsheet.

Video links from the platform YouTube were downloaded with the software Mozdeh^2^ using an Application Programming Interface and the search queries described. Results were combined as described above, randomised, and a sample of 200 videos was obtained.

Videos and news articles were then analysed for the following features: (1) mention of the nine immunological concepts; (2) mention of vaccines or non-pharmacological interventions (NPIs: face masks, lockdown, social distancing) and stance (negative, positive or neutral), as well as a range of political and aesthetic signifiers.

### Analysis of news and video

In coding news articles and videos for their stance on guidelines and vaccines, we considered a negative stance one which would go against the national guidelines. We decided to consider neutral those videos/news that had a negative view on mandatory vaccination but not a negative stance on vaccines, because making vaccinations mandatory or not is part of the implementation of national vaccination strategies and was followed only by some countries. We coded as “anergy/immunoparalysis” videos that stated that COVID-19 vaccination increased susceptibility to infection at later time points.

While Italian videos were all from Italy (none were from Switzerland) those in English were from different geographical locations (the main ones being: United States, 53%, United Kingdom, 9%; Canada, 9%; India, 8% South Africa, 3%; Pakistan, 3%). MediaCloud is a curated database and we specifically searched for news outlets from the United States or Italy.

### Statistical analysis

Correlation of the mention of immunological concepts and a negative stance on different interventions was calculated using a two-tailed, nonparametric Spearman correlation using GraphPad Prism. Results are reported as a correlation matrix showing the Spearman r and interpreted as follows: r is near ±1, perfect correlation; r between ±0.50 and ± 1, strong correlation; r between ±0.30 and ± 0.49, medium correlation; r below ±0.29, low degree of correlation.

As the assessment on the stance (negative, positive or neutral) on vaccines and NPIs is subjective, a sample of news/videos was analysed by two raters to calculate the percentage agreement and weighted Cohen’s kappa using GraphPad prism. Inter-rater reliability was: US news (*n* = 29, raters HG, PG), 88% agreement, kappa = 0.814; Video in English (*n* = 30, raters RSG, HG) 79% agreement, kappa = 0.769; Italian news (*n* = 28, raters MAR, RT), 93% agreement, kappa = 0.880; Italian videos (*n* = 24, raters MAR, RT), 85% agreement, kappa = 0.821. An interpretation of Cohen’s kappa is: Kappa <0, no agreement; 0.00–0.20, slight agreement; 0.21–0.40, fair agreement; 0.41–0.60, moderate agreement; 0.61–0.80, substantial agreement; 0.81–1.00, almost perfect agreement (38).

## Results

### Immunological concepts in media

Table 2 shows the number of hits returned by a search for each of the immunological concepts using the search strategy described in the Methods section (Please note, Table 2 shows only number of hits returned by a search for the keywords as described in the methods and some of these videos or news may not actually mention that specific concept). In the whole MediaCloud database for US news there was a very high number of articles on “herd immunity,” followed by “cytokine storm,” while in the Italian news the distribution was more spread, with “ADE” being the second most frequent concept both in news articles and in videos. Of note, some concepts had few mentions, with some not reaching our target of 25 media for each search to include in the analysis. In the videos returned from a search on YouTube, “immunity debt” was almost absent in Italian, where “herd immunity” was the most frequent concept, while in English-language videos it reached a significant number. It should be noted that not all the articles returned from a search on a specific immunological concept actually mentioned it, and this was even more of an issue with video where it is possible that the search within YouTube returned a video where the search term was only present in some of the comments.

**Table 2:** Mention of different immunological concepts.

Concept	USA news	Italian news	English-language videos	Italian video
ADE	100	459	453	557
Exhaustion/anergy	22	173	999	376
Cytokine storm	1,235	380	494	429
Herd Immunity	15,426	193	934	973
Hygiene Hypoth	25	14	387	15
Immunity Debt	9	27	199	1
OAS	28	35	474	73
Oxidative Stress	107	624	478	488
Viral Interference	18	35	352	18

Data indicating the number of hits returned by MediaCloud (news) or YouTube (video) searching for a specific immunological concept. ADE, antibody-dependent enhancement; OAS, original antigenic sin.

### Prevalence of anti-guideline information

As shown in Table 3, there was a significantly higher prevalence of vaccine-negative information in video than in the news (8-fold in English-language, 6-fold in Italian), where videos negative about vaccines were more numerous than those negative about any NPIs considered. Videos in English had a significantly higher proportion of vaccine-negative information compared to US news (18% vs. 0.9%, *p* > 0.0001 by Chi-square test); Italian videos had a significantly higher proportion of vaccine-negative information compared to Italian news (25% vs. 4%, *p* < 0001 by Chi-square test) or to videos in English (25% vs. 8%, *p* < 0001 by Chi-square test).

**Table 3:** Anti-guideline information in different media.

Negative for:	USA news	Italian news	English videos	Italian videos
Vaccine	2 (0.9%)	8 (4.2%)	18 (8%)	46 (25.1%)
Masks	6 (2.8%)	3 (1.6%)	4 (1.8%)	11 (6.0%)
Lockdown	5 (2.4%)	5 (2.6%)	6 (2.7%)	7 (3.8%)
Distancing	3 (1.4%)	2 (1.1%)	5 (2.2%)	4 (2.2%)
At least one negative	9 (4.2%)	13 (6.8%)	25 (11%)	49 (26.8%)
Total sample	212 (100%)	190 (100%)	225 (100%)	183 (100%)

This was not observed with the stance on various NPIs (a mask-negative stance was slightly more frequent in Italian videos than news but the difference, *p* = 0.02, was not statistically significant after correcting for multiple comparisons). Overall, the prevalence of vaccine-negative videos was three-times higher in Italian than in English.

Number of views had a large variability among the videos analysed and there was no significant difference between guideline-negative videos and the remaining ones. In English, video views were (mean ± SD): guideline-negative, 12,535 ± 32,165 (*n* = 24); other videos 38,573 ± 141,664 (*n* = 201). In Italian: guideline-negative 9,558 ± 20,151 (*n* = 44); other videos 13,303 ± 40,240 (*n* = 139).

### Pattern of mention of anti-guideline information, immunological concepts and “information bubbles”

**Figure 1:**
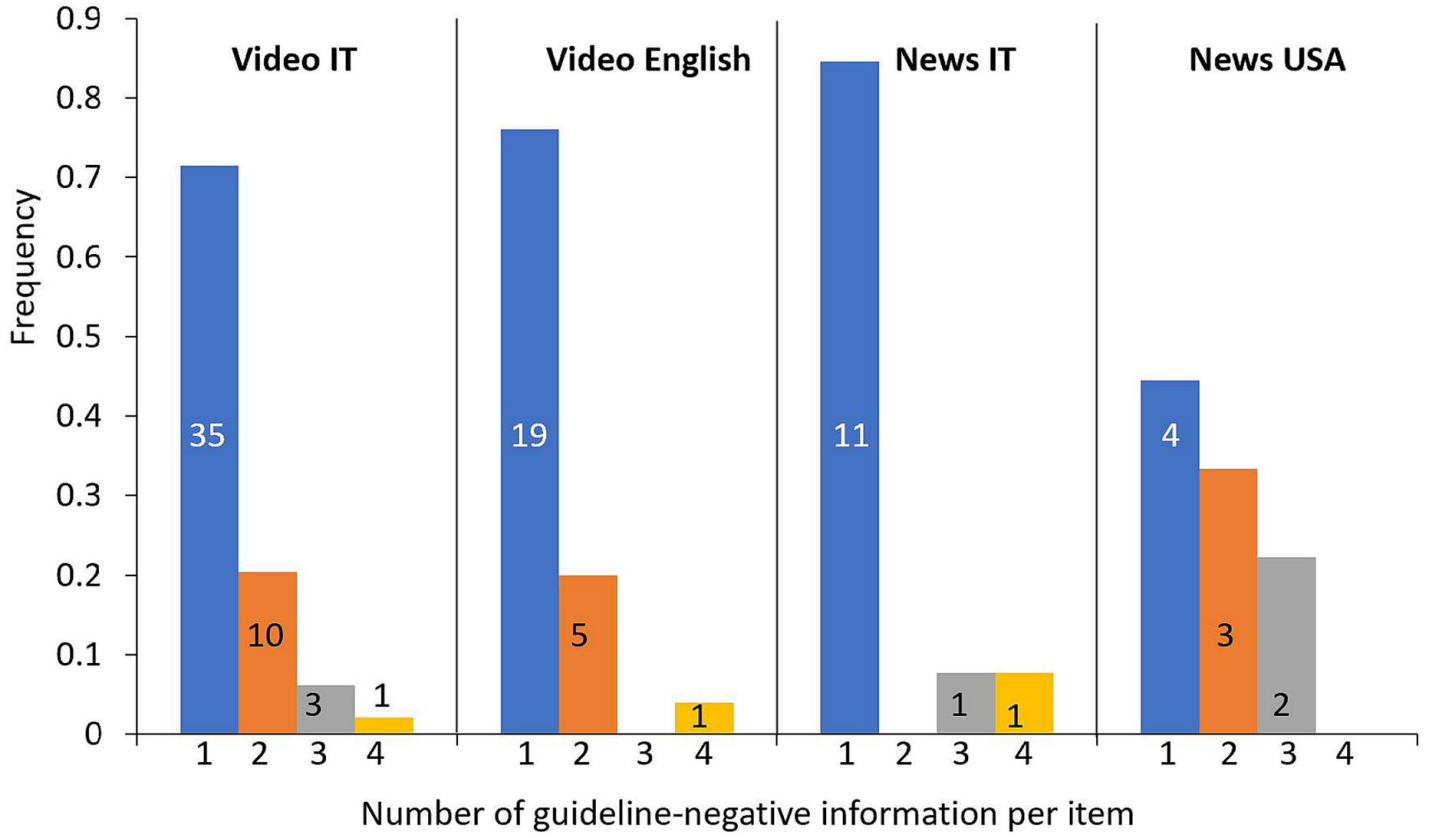
Frequency distribution of videos by the number of interventions for which they have a negative stance. Horizontal axis: number of guideline-negative content per video/news. The numbers of video/news in each group is indicated on the bars.

Another aspect of health misinformation is the “rabbit hole” effect, where interest in unconventional views/media leads to ever more extreme algorithmic recommendations, something which has been observed during COVID-19. We thus looked at whether a negative stance for one intervention was associated with a negative stance towards other interventions. As shown in Figure 1, although most sources had a negative stance for only one intervention, some of them had a negative stance for multiple interventions, and this was particularly evident in US news, where 55% had a negative stance on multiple interventions.

We then explored whether specific scientific concepts were preferentially associated with specific narratives against some interventions, and if a negative view on one intervention was associated with a negative view on another intervention, as suggested by the results shown in Figure 1.

**Figure 2:**
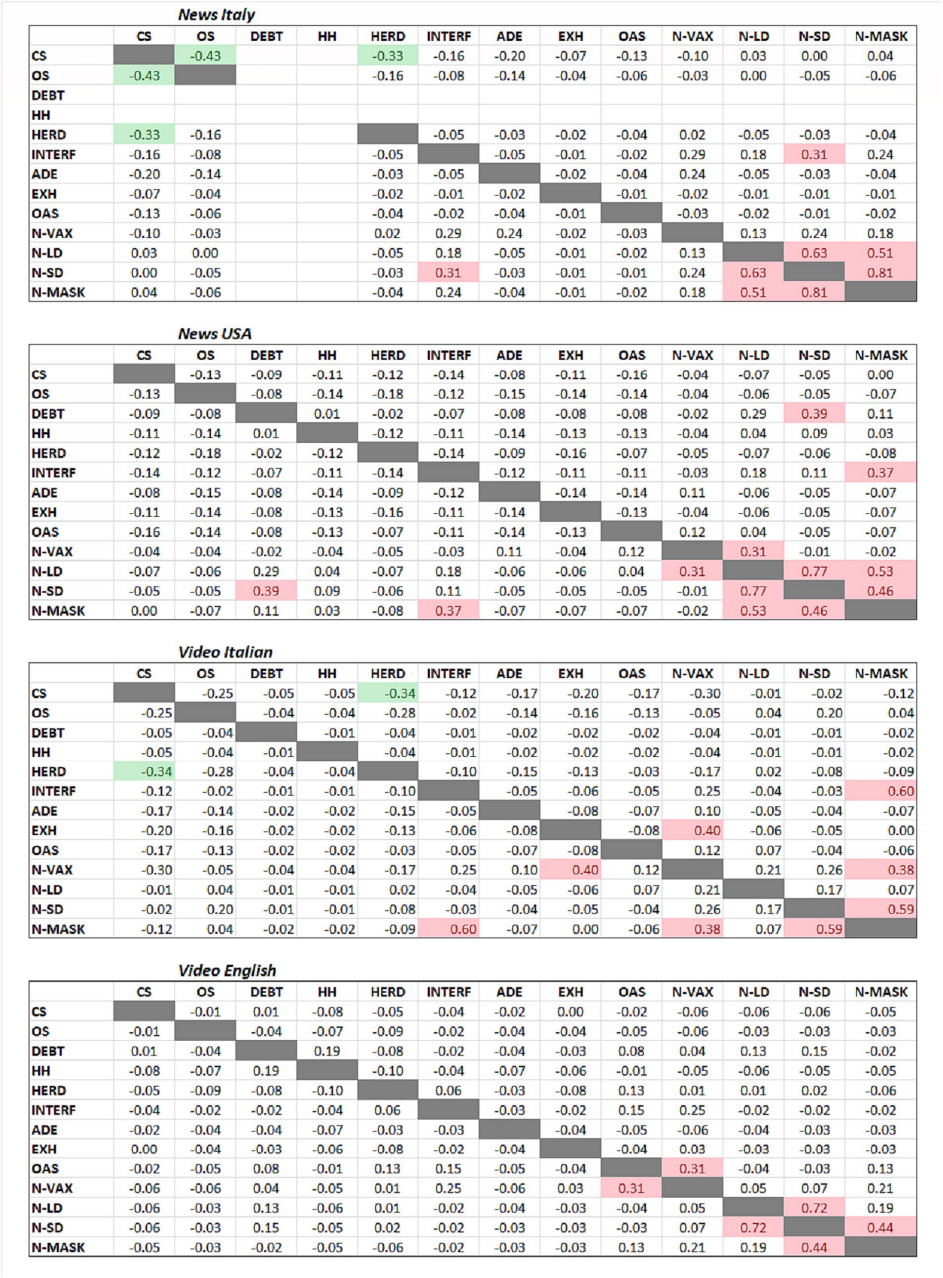
Correlation of mention of immunological concepts and negative (N) view on guidelines. The Spearman r is reported. Red highlights, *r* > 0.3; green, *r* < −0.3. Abbreviations: ADE, antibody-dependent enhancement; CS, cytokine storm; DEBT, immunity debt; EXH, exhaustion/anergy/immunoparalysis; HERD, herd immunity; HH, hygiene hypothesis; INTERF, viral interference; OAS, original antigenic sin; OS, oxidative stress; VAX, vaccine; LD; lockdown; SD, social distancing.

To do this, we looked at the correlation between mention of immunological concepts and negative view of different intervention by calculating the Spearman’s correlation coefficient and visualising the data as a correlation matrix, as shown in Figure 2.

The analysis confirms what was discussed earlier: that in many news/videos, a negative stance for one health intervention was associated with a negative stance on other interventions (note the clusters of correlation on the bottom right of the correlation matrices).

When we look at the association between mention of specific immunological concepts and guideline-negative stances, we found the following (using a Spearman’s r arbitrary cut-off of 0.3): (1) Immunity debt was associated with negative views of social distancing (news-US); (2) Viral interference was associated with negative views of social distancing (video-Italian) and of masks (news-US, video-Italian); (3) Lymphocyte exhaustion/anergy was associated with negative views of vaccines (video-Italian); (4) Original antigenic sin was associated with negative views of vaccines (video-English).

**Figure 3:**
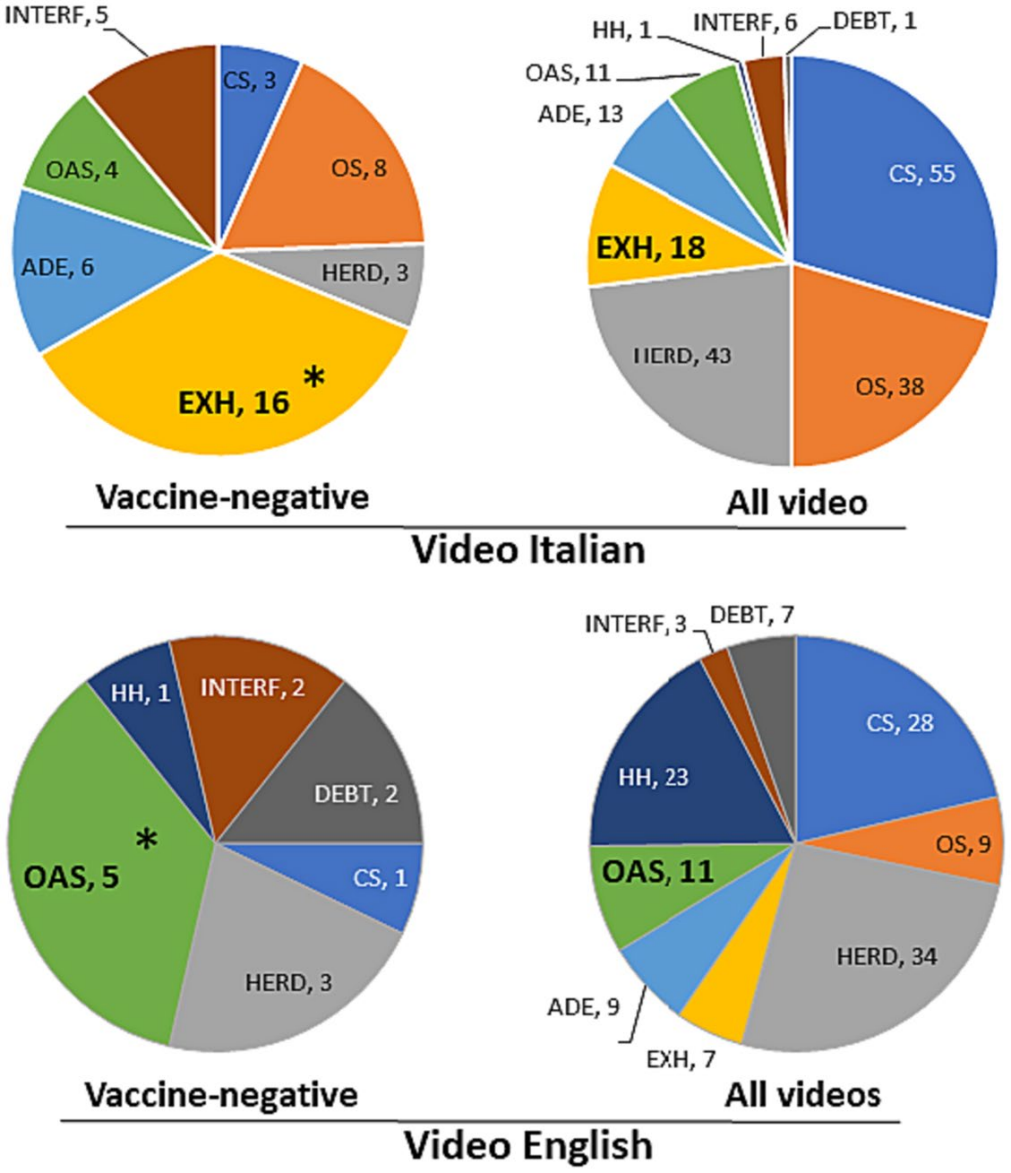
Frequency of mention of immunological concepts in vaccine-negative videos (left) and in the entire sample (right). Number of news/videos mentioning each concept are indicated. Videos may mention more than one immunological concept; those not mentioning any immunological concept are not included in the chart. *Significantly higher in vaccine-negative news/videos. Statistical significance was calculated comparing each negative group with the remaining media (not with all media to avoid comparing overlapping data) with a two-tailed Chi Square test followed by Bonferroni correction for multiple (nine) comparisons (*p* < 0.001).

Of the associations identified above, we analysed in depth those related to a vaccine-negative stance. As shown in Figure 3, frequent mentions of exhaustion in YouTube videos in Italian with a negative view on vaccines was 36% compared to 10% in the whole sample; in English, original antigenic sin was mentioned by 26% of the vaccine-negative video compared to 5% in the whole sample.

## Political bias

**Figure 4:**
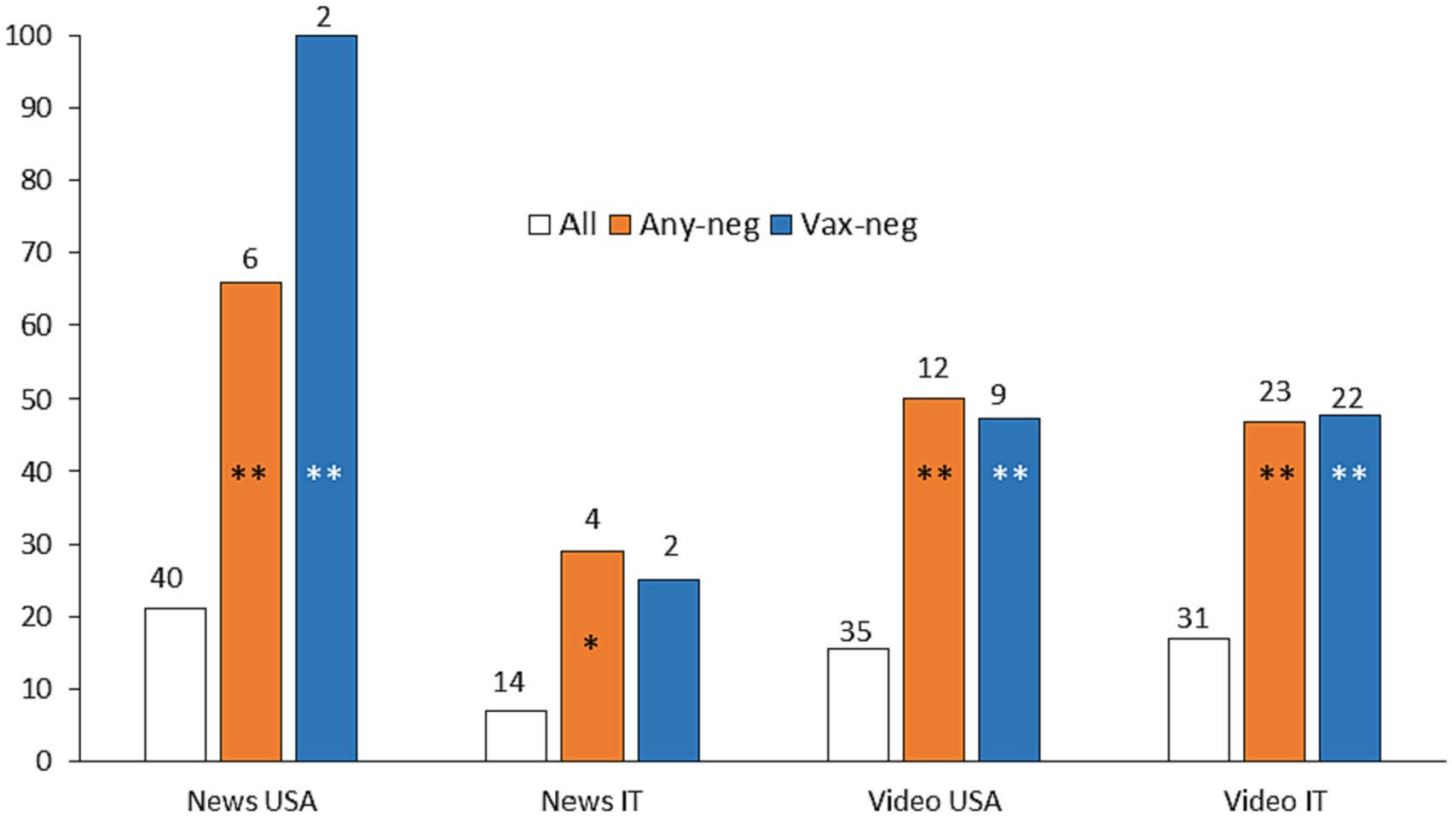
Mention of politics in anti-guideline information. Bars indicate the percentage of information mentioning politics in total news/video (white bars) and in those with a stance negative towards vaccines (blue bars) or at least one intervention (orange bars). The total number of news/video with political mention in each group is shown above the bars and indicates the number of samples on which the percentage was calculated. Statistical significance was calculated comparing each negative group with the remaining media (not with all media to avoid comparing overlapping data) with a two-tailed Chi Square test (*p < 0.05; **p < 0.001).

Politics, either mention of a political view or a politician, was present in several of the media analysed. Figure 4 shows the proportion of news/video with at least one anti-guideline stance or with a vaccine-negative stance mentioning politics. Overall, there were no marked differences in the percentage of media mentioning politics (defined as mention of politics or politicians) among the various media, with Italian news being the least politicised (news IT, 7%; news US, 21%; video IT, 17%, video English, 16%). It is clear that, in all media and languages, politics comes up more frequently in anti-guideline and anti-vaccine media by a factor of 3 in video and of 3–5 in news. It should be noted, however, that, despite the difference being statistically significant, the amount of anti-guideline information in news was very low (with only two vaccine-negative news articles in both languages), which caution about drawing generalised conclusions.

**Figure 5:**
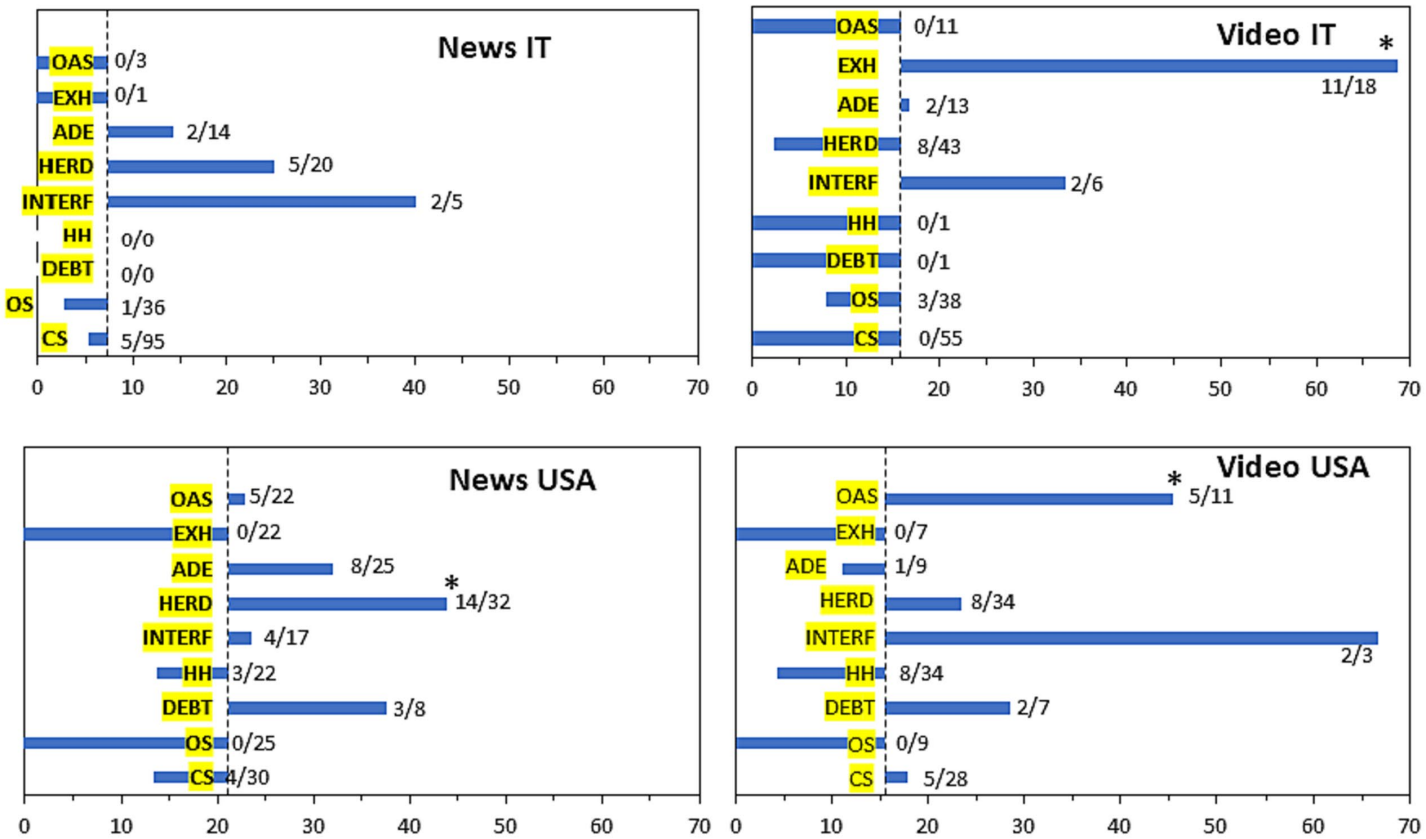
Prevalence of politics in news/video mentioning specific immunological concepts. Data indicate the observed percentage of news/videos on a specific immunological concept that mention politics. The number of samples with political mention/total samples mentioning each concept is shown next to the bars. The dotted line indicates the prevalence of politics in the entire sample, considered the expected value (news IT, 7%; news USA, 21%; video IT, 17%, video English, 16%). Therefore, a bar on the right of the dotted line denotes a prevalence of politics higher than expected for news mentioning that immunological concept; one on the left, a lower than expected politicisation. *Statistically significant over-representation of politics (*p* < 0.01) calculated comparing each negative group with the remaining media (rather than with all media, to avoid comparing overlapping data) by a two-tailed Chi Square test followed by Bonferroni correction for multiple (nine) comparisons. Abbreviations: ADE, antibody-dependent enhancement; CS, cytokine storm; DEBT, immunity debt; EXH, exhaustion/anergy/immunoparalysis; HERD, herd immunity; HH, hygiene hypothesis; INTERF, viral interference; OAS, original antigenic sin; OS, oxidative stress.

We then performed a sub-analysis for the different immunological concepts. Figure 5 shows the political bias in the news mentioning different immunological concepts, where concepts that have an observed presence of politicisation higher than the expected one (their prevalence in the whole sample) lay on the right side of the chart. Of note, this figure also reports the number of news articles in each bar as, in many cases, bars represented very few articles and it is important to avoid over-interpretation of the differences shown.

In the Italian videos, “immunoparalysis/exhaustion/anergy” was significantly polarised while in English-language videos this was the case for “original antigenic sin.” While there was a trend for other concepts being polarised, such as “viral interference,” their small representation in our sample made it impossible to draw any conclusion due to lack of statistical power. “Herd immunity,” one of the most frequently mentioned immunological concepts, did not show any politicisation, except for US news where there was a significant association with politics (14 of 32 articles). Most of these articles (nine) mentioned herd immunity as being potentially achieved with vaccination or (five) describing negatively the initial approach in the United Kingdom and Sweden to achieve herd immunity through infection, at a time where vaccines were not yet available. None of these articles had a negative stance towards vaccination or any of the guidelines.

Further content analysis of videos in English show that within the anti-guideline videos there was a clear bias towards conspiratorial, right-wing ideology, including low government interference, Christian nationalism and evangelical values with 33% espousing those views. Selected quotes from these videos are shown in Supplementary Table S1. This was not observed within the rest of the videos, where 197 of 201 videos had no political bias.

In Italian, 11 of the 29 videos mentioning politics were associated with the political party “Italexit,” mostly by Dr. Frajese, a medical doctor and parliamentary candidate for the party in the 2022 national elections. Italexit advocates the exit of Italy from the European Union and obtained 1.9% of votes in 2022. Unlike videos in English, these video did not have a “libertarian” view but focus on the claim that COVID-19 vaccines are unsafe, and that even the influenza vaccine would increase COVID-19 infections by blocking viral interference, or that the excessive immune stimulation by vaccines would cause immune exhaustion. Transcripts of selected videos are provided in Supplementary Table S2.

## Gender of video speakers

In the English language videos, the proportion of male speakers only was higher in the content with at least one anti-guideline stance (71%) than in the total sample (57%), although the difference was not statistically significant (*p* = 0.19 by a Chi-square test). A similar trend was observed in Italian video, with 67% of male speakers in anti-guideline video and 61% in the whole sample.

## Citation of scientific studies

We also investigated whether news articles had a reference (in the form of a citation or a link) to a scientific publication. Not surprisingly, probably because most scientific publications are in English, only 13 news articles in Italian cited a scientific study. In US news, 64 (34%) cited a scientific study. One of the most cited (five times) was a 2020 study on viral interference between rhinovirus and influenza (39); this study was cited in the context of “viral interference” and only one of the citing news articles had a NPI-negative stance. The second most cited scientific paper (four times) was a study on the potentially negative effect of “immune imprinting” on infection by different SARS-CoV-2 strains (40); none of the citing news articles had an anti-guideline stance.

Table 4 shows how many of the US news articles mentioning each of the immunological concept cited a scientific study. Because often a news article was not focused on the specific immunological concept, we assessed how many citations were actually related to that immune concept. It can be seen that the concept of “viral interference” was associated with the highest number of on-topic citations (82% of the articles cited a scientific paper), followed by “oxidative stress” with 56%; one of these news described an industry-sponsored conference discussing the possible use of N-acetylcysteine,^3^ while a second article included a link to the antioxidant effects of cannabinol, although this was not mentioned in the context of COVID-19.^4^

**Table 4:** Scientific publications cited in USA news mentioning immunological concepts.

Concept	Total news	With citations	On-topic citation
Cytokine storm	30	13	3 (10%)
Oxidative Stress	25	25	14 (56%)
Immunity debt	8	7	3 (38%)
Hygiene hypothesis	22	19	6 (27%)
Herd immunity	32	6	2 (6%)
Viral interference	17	18	14 (82%)
ADE	25	20	8 (32%)
Anergy	16	5	4 (18%)
OAS	22	16	8 (36%)

Data indicate the number of news articles citing a scientific publication in each subgroup. On-topic citations indicate how many of the citations were actually related to the immunology concept.

Of the nine news articles with a negative stance on at least one NPI, four cited scientific publications, most of these supporting the concepts that hygiene is ineffective or even be associated with increased susceptibility to infections, including by preventing viral interference (39, 41–43). One study claimed that fomites may not be important in SARS-CoV-2 transmission (44), while another study described the potential *in vitro* antiviral activity of ivermectin (45).

Only a small proportion of Italian news articles (9 of 190) or Italian videos provided a link or a citation to a scientific article: too few to analyse.

## Discussion

### Presence of anti-vaccine and anti-guideline information in news articles and videos

Our study indicates that negative views on vaccines and mitigation measures were far more frequent in YouTube videos than in newspaper articles, with the proportion of anti-guideline videos particularly high in Italian content. This is not surprising considering anyone can produce and upload a video – there are no “due impartiality” principles as there are for, for example, UK broadcasters (46). Despite this, the proportion of anti-guidelines videos in English (11%) found was less than half as many as in other similar studies on scientific misinformation: a 2010 study of 142 videos on H1N1 influenza found 23% misleading (47), a 2018 study of 101 videos on Zika virus found 23.8% misleading (48), and a 2015 study on 118 videos on Ebola found 26% misleading (49). This could be due to the lower numbers analysed by other studies (indicating a strength of our data), but it also points towards a growing understanding of the dangers of online misinformation, and the success (albeit limited) of YouTube’s guidelines over several years to address issues around child safety and various types of harmful or abusive content (50, 51). Looking at the dates published more closely, however, it seems the most recent efforts, in September 2021, to reduce anti-vaccination content on the platform (52) has not achieved its goals, at least within our sample of videos (data not shown). Anti-vaccination views were the most prominent of the four anti-guideline criteria, with 12 of the 14 misinformation videos post-YouTube guideline introduction being classed as anti-vaccination, whereas only six of the preceding 10 shared those views. This implies that the September 2021 guidelines alone were not effective in their goal. With regards news articles, the proportion of those with an anti-vaccine stance is similar in English and in Italian, albeit slightly lower in English. This is interesting if we consider that the press in Italy is regulated by a professional board (journalists need to be licenced by the Ordine dei Giornalisti) as are UK journalists (by the Independent Press Standards Organisation). In the US journalism is largely unregulated, in accordance with the First Amendment to the US Constitution and after the Federal Communications Commission, under the Reagan administration, repealed the “Fairness Doctrine” in 1987 (53).

The higher proportion of anti-vaccine information in Italian (three times that of videos in English; four times in Italian news compared to the US) raises the question of their impact since, as of 14/9/2022 – end date in our search, the share of the population who were fully vaccinated was not lower in Italy (81%) compared to the United Kingdom (75%), Australia (83%) or the United States (68%) (54).^5^ It is thus possible that the impact of media on the uptake of COVID-19 vaccines is overestimated.

We should also acknowledge that there is an abundant literature on the cultural differences in health communication, reflecting a different traditional media market (55, 56) and studies on communication of the COVID-19 pandemics have highlighted the different weight given to political actors across different countries (57). Additionally, there are cultural differences in the information seeking behaviour of the public (58) and in the susceptibility to misinformation, as shown in the case of COVID-19 (59). Therefore, the results presented here should be considered as only one of the aspects of the problems associated with health information and science communication in general.

### Information bubbles and immunological concepts

The analysis of possible “information bubbles” confirmed, in all type of media analysed, that readers/viewers exposed to information with a negative stance on one public health measure are often exposed to negative stances on other measures, too, which may contribute to the polarisation of these issues – a phenomenon that has been reported by others. While social media and the Internet are often blamed for the formation of information bubbles (60), this also suggests a contagious transmission from one conspiracy theory to another (61), described by Meyer as “social spill-over” (62).

Looking at whether immunological concepts were used to bolster the authority of the anti-guideline videos, it is clear that during the pandemic the global community expanded its vocabulary significantly with new phrases about virology, vaccinology and epidemiology (63). Within the anti-guideline videos these concepts were used in different ways. Some did not seem to have caught the attention of the YouTubers producing this content: antibody-dependent enhancement, oxidative stress and anergy were not mentioned. Cytokine storm tended to be described and used in an accurate way. Herd immunity was decried as either impossible to achieve or antithetical to a free way of living – a desire not to be a member of a herd. Hygiene hypothesis and immunity debt similarly seemed to trigger culture war-like comments on children being affected long term by lack of natural immunity, or our sanitised, modern way of living (linked to masks, distancing, and especially lockdowns) damaging us far more than a Covid-19 infection would. Our analysis did show that some immunological concepts, particularly those of immune exhaustion/lymphocyte anergy and original antigenic sin/immune imprinting were mis-used in anti-vaccine discourse, however.

Exhaustion, anergy and activation-induced cell death are well-known concepts described in all immunology textbooks and represent mechanisms mainly aimed at preventing autoimmunity, known as central and peripheral tolerance (64). However, they can also occur when lymphocytes are stimulated over a prolonged period, such as in chronic infection (65, 66). Promoters of anti-vaccine views often warn that repeated vaccination may cause immune exhaustion so that subsequent boosters would not provide additional protection and would dampen the immune response. This hypothesis is clearly refuted by many studies on the effectiveness of additional COVID-19 vaccine boosters (67–69), not to mention the efficacy of the many vaccines given to newborns in the first years of life.

Original antigenic sin (also immune imprinting or antigen imprinting) was first postulated in 1960 in relation to influenza (70) and describes the fact that the most efficient response mounted by the immune system against infections is that against the first variant of a pathogen already encountered in life. The evolutionary reason for this is that, often, conserved antigens are the ones most important for the pathogen (36). This concept has also been used in anti-vaccine information to minimise the effect of vaccines in the presence of a mutating virus or by claiming that vaccines would increase susceptibility to subsequent mutants. While the original antigenic sin described for COVID-19 and infection with SARS-Cov-2 elicit responses to coronaviruses encountered earlier in life, particularly the seasonal human coronaviruses (HCoVs) that cause common cold (37, 71), this seems not to impact the efficacy of COVID-19 vaccines and boosters (72).

Another concept that we found associated with anti-guideline information is that of “immunity debt.” This has been used to support the hypothesis that COVID-19 mitigation measures such as lockdowns and social distancing or the use of face masks cause an increase in later respiratory infections. The popularity of this concept is likely due to the fact that it can be easily understood by the public; nevertheless, there is no mention of it in formal immunology textbooks. To date, it is mentioned in only 15 scientific publications found in PubMed, of which 13 are studies on reviews, editorials, letter, studies of health communication or mathematical models, and three are studies reporting a rise in the incidence of paediatric infections after the main pandemic peak, hypothesising immunity debt as a possible explanation.

Concepts like “oxidative stress” (25, 73) and “cytokine storm” (74) are not controversial as they merely represent potential pathogenetic mechanisms, so were often negatively associated with politicisation. Of note, while the role of oxidative stress in COVID-19 remains largely hypothetical, the cytokine storm theory has been successfully applied to the field, with the approval of interleukin-6 inhibitors for COVID-19 (75).

The use of scientific papers was demonstrated in a similar way across all videos: the power of the phrase “studies have shown” (76) being used to reinforce points in anti-and pro-guideline content. This highlights the tricky nature of citing scientific research within communications to lay people (77). Most members of the public would not follow up a claim in a video by searching out and reading the paper cited, but they might feel the claim is more legitimate due to the mention. For example, on Sky News, Rita Panahi cited “a French study” on immunity debt. She was trying to use the research to imply that lockdowns were fundamentally dangerous and damaging to health, especially that of children. The study she was likely referring to [Cohen et al., although she did not mention it specifically (78)] was far more balanced than her portrayal, however, acknowledging the importance of not overloading the healthcare system, and couching their opinions about the future consequences of a lack of immune stimulation using phrases such as “could have negative consequences” or “greater the likelihood” (78). This illustrates a trend within misinformation videos to use the equivocal scientific language style as a reason to doubt, or to question scientific evidence (32). There are rarely absolutes in scientific theory: that is the nature of research, but this can be exploited by those willing to misinterpret data for their own aims (33).

### Political and ideological issues

We found that politicisation (identified in this study as a mention of political issues or politicians) is higher in news/videos purporting anti-vaccine or anti-NPI views. Interestingly, politicisation is over-represented in videos mentioning the two immunological concepts exhaustion/anergy and original antigenic sin, that are also associated with anti-vaccine stances.

The qualitative analysis part of our study confirms the association between far-right political stances and anti-vaccine views that has been reported in many countries (79–85), although it in Italy an anti-vaccination stance may also be associated with some far-left activists (86, 87).

In videos in English, it was clear how overtly right wing many of the anti-guideline videos were, compared to the rest. Right ideology including low government interference, small government, social conservatism, rejection of socialism in support of capitalism and neoliberalism, antipathy towards the media and anti-globalism were oft-cited.

There is also power in centring anecdotal evidence within a narrative of scientific language and right-wing ideology, such as seen by Dr. Robert Malone – he combines all three ideas within one sentence: “Talk to the people around you... it’s called immune imprinting...what the government has bought is the perfect vaccine combination to cause this to get even worse.”^6^ With regards to drugs, both approved and experimental, our theory was that ivermectin and hydroxychloroquine, both medications promoted by the far right as effective against Covid, would be regularly mentioned. This was not the case.

We also noted that videos had more often a male communicator than a female and this has also been observed by other studies of science communication on YouTube (88) Interestingly, we found that this gender difference was even more evident in videos negative about guidelines. Studies in the United States have reported that masculinity is a predictor of lower adherence to the Centers for Disease Control and Prevention (CDC) guidelines for the prevention of COVID-19 (89, 90). However, the gender bias is not only found at the receiving end (the consumer of information). An experimental study on the gender of scientists mentioned in news articles on COVID-19 vaccines has shown that the presence of a female inventor, either by herself or together with a male inventor, had a negative effect on how safety and efficacy of the vaccine was perceived (91).

This reflects wider voting patterns between right and left wing choice – men tend to choose right wing politics, and tend to reject health advice (e.g., wearing sun protection, cycle helmets) (92–94). This even extends to families – having daughters results in parents voting for left wing parties, but having sons reverses this trend (95). It also highlights a growing antipathy towards feminist values within right wing ideology (26, 96), with openly chauvinist groups growing in public prominence, and Christian fundamentalist nationalism emerging as a powerful wing of mainstream US politics (97). The language of religion is even combined with the immunological concepts in several videos, as the presenters mock the vaccination programme: “It’s called original antigenic sin, which is just kind of really, wow, yeah, the scientists aren’t, you know, completely illiterate about matters of faith.” (Vaccine Information for Catholics with Pam Acker, United States).^7^

## Conclusion

This study shows it is clear that some YouTubers are using immunological concepts to further their anti-guideline agendas and make themselves appear more knowledgeable and authoritative, however these concepts are very selective and limited, dependent on their interpretability by lay viewers. The significance of this is that it shows the usefulness of certain immunological terms as a way to distinguish and remove dangerous content from online platforms, albeit in a limited way. It could be important that international and regional health authorities address this issue in their educational material, by explaining these concepts to the public and putting them in the correct scientific context. Our study also shows the importance of more widespread scientific literacy across the general public, such as an understanding of how to read and interpret a basic scientific paper; this type of education could disarm the use of studies as a way to bolster credibility. Our findings also reinforce the long-established gender bias within right wing media, while also illustrating the symbiosis of right wing, conspiracist and religious movements fuelling the Covid-19 infodemic.

However, although we used a sample size of nearly 800 videos/news articles, our study still lacks statistical power in analysing concepts only mentioned in a few videos. Additionally, the low proportion of anti-vaccine news articles was also a limitation. Clearly, studies on larger samples are needed and, because this may not be feasible for human raters, automated methods relying on natural language processing may be needed. Another limitation of this study is that restricting the analysis to news articles and YouTube ignores social media used by younger generations, such as Snapchat, Instagram or TikTok (98).

## Data availability statement

Publicly available datasets were analysed in this study. This data can be found at: https://zenodo.org/doi/10.5281/zenodo.8382702.

## Author contributions

RG: Conceptualization, Data curation, Formal analysis, Investigation, Methodology, Writing – original draft, Writing – review & editing. HG: Conceptualization, Data curation, Formal analysis, Investigation, Methodology, Writing – original draft, Writing – review & editing. MR: Conceptualization, Data curation, Formal analysis, Investigation, Methodology, Writing – original draft, Writing – review & editing. RT: Conceptualization, Data curation, Formal analysis, Investigation, Methodology, Writing – original draft, Writing – review & editing PG: Conceptualization, Data curation, Formal analysis, Investigation, Methodology, Project administration, Supervision, Writing – original draft, Writing – review & editing.

## Footnotes

^1^ www.MediaCloud.org

^2^ http://mozdeh.wlv.ac.uk

^3^ https://www.thestreet.com/press-releases/30th-quot-european-respiratory-society-quot-congress-15423544

^4^ https://reason.com/2022/01/28/instagram-bans-healthy-eating-ads-for-promoting-negative-self-perception/

^5^ https://ourworldindata.org/covid-vaccinations

^6^ YouTube.com/watch?v=eDUfwSUZRDA

^7^ YouTube.com/watch?v=TaAUnmtqCNk

## References

[1] Krawczyk K Chelkowski T Laydon DJ Mishra S Xifara D Gibert B. Quantifying online news media coverage of the COVID-19 pandemic: text mining study and resource. J Med Internet Res. (2021) 23:e28253. doi: 10.2196/28253, PMID: 33900934 PMC8174556

[2] Capurro G Jardine CG Tustin J Driedger M. Communicating scientific uncertainty in a rapidly evolving situation: A framing analysis of Canadian coverage in early days of COVID-19. BMC Public Health. (2021) 21:2181. doi: 10.1186/s12889-021-12246-x, PMID: 34844582 PMC8628029

[3] Evans R. SAGE advice and political decision-making: ‘following the science’ in times of Epistemic uncertainty. Soc Stud Sci. (2022) 52:53–78. doi: 10.1177/03063127211062586, PMID: 34963397

[4] Fleerackers A Riedlinger M Moorhead L Ahmed R Alperin JP. Communicating scientific uncertainty in an age of COVID-19: an investigation into the use of preprints by digital media outlets. Health Commun. (2022) 37:726–38. doi: 10.1080/10410236.2020.1864892, PMID: 33390033

[5] Parviainen J Koski A Torkkola S. Building a ship while sailing it. Epistemic humility and the temporality of non-knowledge in political decision-making on COVID-19. Soc Epistemol. (2021) 35:232–44. doi: 10.1080/02691728.2021.1882610

[6] Cinelli M Quattrociocchi W Galeazzi A Valensise CM Brugnoli E Schmidt AL. The COVID-19 social media Infodemic. Sci Rep. (2020) 10:16598. doi: 10.1038/s41598-020-73510-5, PMID: 33024152 PMC7538912

[7] Koffman J Gross J Etkind SN Selman L. Uncertainty and COVID-19: how are we to respond? J R Soc Med. (2020) 113:211–6. doi: 10.1177/0141076820930665, PMID: 32521198 PMC7439590

[8] Larsen EM Donaldson KR Liew M Mohanty A. Conspiratorial thinking during COVID-19: the roles of paranoia, delusion-proneness, and intolerance of uncertainty. Front Psych. (2021) 12:698147. doi: 10.3389/fpsyt.2021.698147, PMID: 34483993 PMC8416269

[9] Valerie VM Pummerer LJ Alper S Bai H Čavojová V Farias J. Antecedents and consequences of COVID-19 conspiracy beliefs: A systematic review. Soc Sci Med. (2022) 301:114912. doi: 10.1016/j.socscimed.2022.114912, PMID: 35354105 PMC8920084

[10] Bertin P Nera K Delouvée S. Conspiracy beliefs, rejection of vaccination, and support for hydroxychloroquine: A conceptual replication-extension in the COVID-19 pandemic context. Front Psychol. (2020) 11:565128. doi: 10.3389/fpsyg.2020.565128, PMID: 33071892 PMC7536556

[11] Lazarević LB Purić D Teovanović P Lukić P Zupan Z Knežević G. What drives us to be (Ir)responsible for our health during the COVID-19 pandemic? The role of personality, thinking styles, and conspiracy mentality. Personal Individ Differ. (2021) 176:110771. doi: 10.1016/j.paid.2021.110771, PMID: 33612906 PMC7879160

[12] Bozeman B. Use of science in public policy: lessons from the COVID-19 pandemic efforts to ‘follow the science. Sci Public Policy. (2022) 49:806–17. doi: 10.1093/scipol/scac026

[13] Cairney P. The UK Government’s COVID-19 policy: what does ‘guided by the science’ mean in practice? Front Political Sci. (2021) 3:624068. doi: 10.3389/fpos.2021.624068

[14] MacAulay M Fafard P Cassola A Palkovits M. Analysing the ‘follow the science’ rhetoric of government responses to COVID-19. Policy Polit. (2023) 51:466–85. doi: 10.1332/030557321X16831146677554

[15] Leidecker-Sandmann M Attar P Schütz A Lehmkuhl M. Selected by expertise? Scientific experts in German news coverage of COVID-19 compared to other pandemics. Public Underst Sci. (2022) 31:096366252210957:847–66. doi: 10.1177/09636625221095740, PMID: 35723453

[16] Aelst V Peter FT Castro L Štětka V de Vreese C Aalberg T. Does a crisis change news habits? A comparative study of the effects of COVID-19 on news media use in 17 European countries. Digit Journal. (2021) 9:1208–38. doi: 10.1080/21670811.2021.1943481

[17] Ioannidis JP Tezel A Jagsi R. Overall and COVID-19-specific citation impact of highly visible COVID-19 media experts: bibliometric analysis. BMJ Open. (2021) 11:e052856. doi: 10.1136/bmjopen-2021-052856, PMID: 34706959 PMC8551747

[18] Wormer H. German media and coronavirus: exceptional communication—or just a catalyst for existing tendencies? Media Commun. (2020) 8:467–70. doi: 10.17645/mac.v8i2.3242

[19] Dolman AJ Fraser T Panagopoulos C Aldrich DP Kim D. Opposing views: associations of political polarization, political party affiliation, and social trust with COVID-19 vaccination intent and receipt. J Public Health. (2022) 45:36–9. doi: 10.1093/pubmed/fdab401, PMID: 35077546 PMC9383304

[20] Pinna M Picard L Goessmann C. Cable news and COVID-19 vaccine compliance. Rochester, NY: Social Science Research Network (2021).

[21] Allgaier J. Who is having a voice? Journalists’ selection of sources in a creationism controversy in the UK press. Cult Stud Sci Educ. (2011) 6:445–67. doi: 10.1007/s11422-011-9319-5

[22] Albæk E. The interaction between experts and journalists in news journalism. Aust Dent J. (2011) 12:335–48. doi: 10.1177/1464884910392851

[23] Vaninov N. In the eye of the COVID-19 cytokine storm. Nat Rev Immunol. (2020) 20:277–7. doi: 10.1038/s41577-020-0305-6, PMID: 32249847 PMC7132547

[24] Mangalmurti N Hunter CA. Cytokine storms: understanding COVID-19. Immunity. (2020) 53:19–25. doi: 10.1016/j.immuni.2020.06.017, PMID: 32610079 PMC7321048

[25] Sies H Berndt C Jones DP. Oxidative stress. Annu Rev Biochem. (2017) 86:715–48. doi: 10.1146/annurev-biochem-061516-045037 28441057

[26] Ghezzi P Jaquet V Marcucci F Schmidt HH. The oxidative stress theory of disease: levels of evidence and epistemological aspects. Br J Pharmacol. (2016) 174:1784–96. doi: 10.1111/bph.13544, PMID: 27425643 PMC5446567

[27] Cohen R Pettoello-Mantovani M Somekh E Levy C. European pediatric societies call for an implementation of regular vaccination programs to contrast the immunity debt associated to coronavirus Disease-2019 pandemic in children. J Pediatr. (2022) 242:260–261.e3. doi: 10.1016/j.jpeds.2021.11.061, PMID: 34848191 PMC8626874

[28] Yazdanbakhsh M Kremsner PG van Ree R. Allergy, parasites, and the hygiene hypothesis. Science. (2002) 296:490–4. doi: 10.1126/science.296.5567.490, PMID: 11964470

[29] Bach J-F. The hygiene hypothesis in autoimmunity: the role of pathogens and commensals. Nat Rev Immunol. (2018) 18:105–20. doi: 10.1038/nri.2017.111, PMID: 29034905

[30] Metcalf CJE Ferrari M Graham AL Grenfell BT. Understanding herd immunity. Trends Immunol. (2015) 36:753–5. doi: 10.1016/j.it.2015.10.004 26683689

[31] Piret J Boivin G. Viral interference between respiratory viruses. Emerg Infect Dis. (2022) 28:273–81. doi: 10.3201/eid2802.211727, PMID: 35075991 PMC8798701

[32] Arvin AM Fink K Schmid MA Cathcart A Spreafico R Havenar-Daughton C. A perspective on potential antibody-dependent enhancement of SARS-CoV-2. Nature. (2020) 584:353–63. doi: 10.1038/s41586-020-2538-8, PMID: 32659783

[33] Wherry EJ. T cell exhaustion | nature immunology. Nat Immunol. (2011) 12:492–499. doi: 10.1038/ni.2035 21739672

[34] Wherry EJ Kurachi M. Molecular and cellular insights into T cell exhaustion. Nat Rev Immunol. (2015) 15:486–99. doi: 10.1038/nri3862, PMID: 26205583 PMC4889009

[35] Pillai S. SARS-CoV-2 vaccination washes away original antigenic sin. Trends Immunol. (2022) 43:271–3. doi: 10.1016/j.it.2022.02.009, PMID: 35272935 PMC8882427

[36] Zhang A Stacey HD Mullarkey CE Miller MS. Original Antigenic Sin: How First Exposure Shapes Lifelong Anti–Influenza Virus Immune Responses. J Immunol. (2019) 202:335–40. doi: 10.4049/jimmunol.1801149 30617114

[37] Aguilar-Bretones M Fouchier RAM Koopmans MPG van Nierop GP. Impact of antigenic evolution and original antigenic sin on SARS-CoV-2 immunity. J Clin Invest. (2023) 133:e162192. doi: 10.1172/JCI162192, PMID: 36594464 PMC9797340

[38] Landis JR Koch GG. The measurement of observer agreement for categorical data. Biometrics. (1977) 33:159–74. doi: 10.2307/2529310 843571

[39] Wu A Mihaylova VT Landry ML Foxman EF. Interference between rhinovirus and influenza A virus: A clinical data analysis and experimental infection study. Lancet Microbe. (2020) 1:e254–62. doi: 10.1016/S2666-5247(20)30114-2, PMID: 33103132 PMC7580833

[40] Reynolds CJ Pade C Gibbons JM Otter AD Lin K-M Sandoval DM. Immune boosting by B.1.1.529 (omicron) depends on previous SARS-CoV-2 exposure. Science. (2022) 377:eabq1841. doi: 10.1126/science.abq1841, PMID: 35699621 PMC9210451

[41] Finlay BB Amato KR Azad M Blaser MJ Bosch TCG Chu H. The hygiene hypothesis, the COVID pandemic, and consequences for the human microbiome. Proc Natl Acad Sci. (2021) 118:e2010217118. doi: 10.1073/pnas.2010217118, PMID: 33472859 PMC8017729

[42] Lynch SJ Sears MR Hancox RJ. Thumb-sucking, nail-biting, and atopic sensitization, asthma, and Hay fever. Pediatrics. (2016) 138:e20160443. doi: 10.1542/peds.2016-0443, PMID: 27401101

[43] Xiao J Shiu EYC Gao H Wong JY Fong MW Ryu S. Nonpharmaceutical measures for pandemic influenza in nonhealthcare settings—personal protective and environmental measures. Emerg Infect Dis. (2020) 26:967–75. doi: 10.3201/eid2605.190994, PMID: 32027586 PMC7181938

[44] Ben-Shmuel A Brosh-Nissimov T Glinert I Bar-David E Sittner A Poni R. Detection and infectivity potential of severe acute respiratory syndrome coronavirus 2 (SARS-CoV-2) environmental contamination in isolation units and quarantine facilities. Clin Microbiol Infect: Official Pub European Society of Clin Microbiol Infect Dis. (2020) 26:1658–62. doi: 10.1016/j.cmi.2020.09.004, PMID: 32919072 PMC7481174

[45] Gupta S Sarthi P Biswal S Panda SK Ray AK Rana MK. Binding mechanism and structural insights into the identified protein target of COVID-19 and importin-α with in-vitro effective drug Ivermectin. J Biomol Struct Dyn. (2022) 40:2217–26. doi: 10.1080/07391102.2020.1839564, PMID: 33111618 PMC7605516

[46] Ofcom. (2021). Section five: Due impartiality and due accuracy. Section five: Due impartiality and due accuracy. January 5, 2021. Available at: https://www.ofcom.org.uk/tv-radio-and-on-demand/broadcast-codes/broadcast-code/section-five-due-impartiality-accuracy. Archived at https://web.archive.org/web/20230808113522/https://www.ofcom.org.uk/tv-radio-and-on-demand/broadcast-codes/broadcast-code/section-five-due-impartiality-accuracy.

[47] Pandey A Patni N Singh M Sood A Singh G. YouTube as a source of information on the H1N1 influenza pandemic. Am J Prev Med. (2010) 38:e1–3. doi: 10.1016/j.amepre.2009.11.007, PMID: 20171526

[48] Bora K Das D Barman B Borah P. Are internet videos useful sources of information during global public health emergencies? A case study of YouTube videos during the 2015-16 Zika virus pandemic. Pathogens and Global Health. (2018) 112:320–8. doi: 10.1080/20477724.2018.1507784, PMID: 30156974 PMC6381519

[49] Pathak R Poudel DR Karmacharya P Pathak A Aryal MR Mahmood M. YouTube as a source of information on Ebola virus disease. N Am J Med Sci. (2015) 7:306–9. doi: 10.4103/1947-2714.161244, PMID: 26258077 PMC4525388

[50] Yesilada M Lewandowsky S. Systematic review: YouTube recommendations and problematic content. Internet Policy Rev. (2022) 11:1652. doi: 10.14763/2022.1.1652, PMID: 36466439 PMC7613872

[51] YouTube Progress on Responsibility. (2021). YouTube Progress on Responsibility - How YouTube Works. 2021. Available at: https://www.youtube.com/howyoutubeworks/progress-impact/responsibility/. Archived at https://web.archive.org/web/20230808115733/https://www.youtube.com/intl/ALL_uk/howyoutubeworks/progress-impact/responsibility/.

[52] YouTube Team. (2021). “Managing harmful vaccine content on YouTube.” Blog.Youtube. 2021. Available at: https://blog.youtube/news-and-events/managing-harmful-vaccine-content-youtube/ Archived at https://web.archive.org/web/20230711100633/https://blog.youtube/news-and-events/managing-harmful-vaccine-content-youtube/.

[53] Perlman A. Whitewashing diversity: the conservative attack on the ‘stealth fairness doctrine. Telev New Media. (2012) 13:353–73. doi: 10.1177/1527476411423676

[54] Mathieu E Ritchie H Ortiz-Ospina E Roser M Hasell J Appel C. A global database of COVID-19 vaccinations. Nat Hum Behav. (2021) 5:947–53. doi: 10.1038/s41562-021-01122-8 33972767

[55] Tang L Peng W. Culture and health reporting: A comparative content analysis of newspapers in the United States and China. J Health Commun. (2015) 20:187–95. doi: 10.1080/10810730.2014.920060, PMID: 25411911

[56] Vestergaard GL Nielsen KH. Science news in a closed and an open media market: A comparative content analysis of print and online science news in Denmark and the United Kingdom. Eur J Commun. (2016) 31:661–77. doi: 10.1177/0267323116674110

[57] Zhang W Cheung YL. The hierarchy of news values – A Corpus-based diachronic and cross-cultural comparison of news reporting on epidemics. J Lesbian Stud. (2022) 23:281–301. doi: 10.1080/1461670X.2021.2021104

[58] Lu L Liu J Connie Yuan Y. Health information seeking behaviors and source preferences between Chinese and U.S. populations. J Health Commun. (2020) 25:490–500. doi: 10.1080/10810730.2020.1806414, PMID: 33150861

[59] Kim HK Ahn J Atkinson L Kahlor LA. Effects of COVID-19 misinformation on information seeking, avoidance, and processing: A multicountry comparative study. Forensic Sci Commun. (2020) 42:586–615. doi: 10.1177/1075547020959670 PMC7492825 38603002

[60] Nguyen CT. Echo chambers and epistemic bubbles. Episteme. (2020) 17:141–61. doi: 10.1017/epi.2018.32

[61] Debnath R Reiner DM Sovacool BK Müller-Hansen F Tim Repke R Alvarez M. Conspiracy spillovers and geoengineering. iScience. (2023) 26:106166. doi: 10.1016/j.isci.2023.106166, PMID: 36994188 PMC10040962

[62] Meyer DS Whittier N. Social movement spillover. Soc Probl. (1994) 41:277–98. doi: 10.2307/3096934

[63] Atabekova A Lutskovskaia L Kalashnikova E. Axiology of Covid-19 as a linguistic phenomenon. J Inf Sci. (2022) 50:245–253. doi: 10.1177/01655515221091542

[64] Abbas A Lichtman A Pillai S. Cellular and Molecular Immunology: Cellular and Molecular Immunology E-Book. Netherlands: Elsevier Health Sciences (2021).

[65] Blank CU Nicholas Haining W Held W Hogan PG Kallies A Lugli E. Defining 'T cell exhaustion'. Nat Rev Immunol. (2019) 19:665–74. doi: 10.1038/s41577-019-0221-9, PMID: 31570879 PMC7286441

[66] Mueller SN Ahmed R. High antigen levels are the cause of T cell exhaustion during chronic viral infection. Proc Natl Acad Sci. (2009) 106:8623–8. doi: 10.1073/pnas.0809818106, PMID: 19433785 PMC2688997

[67] Lau JJ Cheng SMS Leung K Lee CK Hachim A Tsang LCH. Real-world COVID-19 vaccine effectiveness against the omicron BA.2 variant in a SARS-CoV-2 infection-naive population. Nat Med. (2023) 29:348–57. doi: 10.1038/s41591-023-02219-5, PMID: 36652990 PMC9941049

[68] Magen O Waxman JG Makov-Assif M Vered R Dicker D Hernán MA. Fourth dose of BNT162b2 mRNA Covid-19 vaccine in a Nationwide setting. N Engl J Med. (2022) 386:1603–14. doi: 10.1056/NEJMoa2201688, PMID: 35417631 PMC9020581

[69] Munro APS Feng S Janani L Cornelius V Aley PK Babbage G. Safety, immunogenicity, and Reactogenicity of BNT162b2 and mRNA-1273 COVID-19 vaccines given as fourth-dose boosters following two doses of ChAdOx1 nCoV-19 or BNT162b2 and a third dose of BNT162b2 (COV-BOOST): A multicentre, blinded, phase 2, randomised trial. Lancet Infect Dis. (2022) 22:1131–41. doi: 10.1016/S1473-3099(22)00271-7, PMID: 35550261 PMC9084623

[70] Francis T. On the doctrine of original antigenic sin. Proc Am Philos Soc. (1960) 104:572–8.

[71] Aguilar-Bretones M Westerhuis BM Raadsen MP de Bruin E Chandler FD Nisreen MA. Seasonal coronavirus-specific B-cells with limited SARS-CoV-2 cross-reactivity dominate the IgG response in severe COVID-19 patients. American Society for Clin Invest. (2021) 131:2021. doi: 10.1172/JCI150613, PMID: 34499051 PMC8553556

[72] Azuma H Kawano Y Shitaoka K Kawahara T Ito A Higashiura A. Vaccination with the omicron spike RBD boosts broadly neutralizing antibody levels and confers sustained protection even after acquiring immunity to the original antigen. Int Immunol. (2023) 35:197–207. doi: 10.1093/intimm/dxac055, PMID: 36413150

[73] Sies H Belousov VV Chandel NS Davies MJ Jones DP Mann GE. Defining roles of specific reactive oxygen species (ROS) in cell biology and physiology. Nat Rev Mol Cell Biol. (2022) 23:499–515. doi: 10.1038/s41580-022-00456-z, PMID: 35190722

[74] Fajgenbaum DC June CH. Cytokine Storm. N Engl J Med. (2020) 383:2255–73. doi: 10.1056/NEJMra2026131, PMID: 33264547 PMC7727315

[75] Recovery Collaborative Group. Tocilizumab in patients admitted to hospital with COVID-19 (RECOVERY): A randomised, controlled, open-label, platform trial. Lancet (London, England). (2021) 397:1637–45. doi: 10.1016/S0140-6736(21)00676-0, PMID: 33933206 PMC8084355

[76] Gorski D Yamey G. Covid-19 and the new merchants of doubt. BMJ. (2021) 13:2021.

[77] Maani N van Schalkwyk MCI Filippidis FT Knai C Petticrew M. Manufacturing doubt: assessing the effects of independent vs industry-sponsored messaging about the harms of fossil fuels, smoking, alcohol, and sugar sweetened beverages. SSM - Population Heal. (2022) 17:101009. doi: 10.1016/j.ssmph.2021.101009, PMID: 35036514 PMC8749266

[78] Cohen R Ashman M Taha M-K Varon E Angoulvant F Levy C. Pediatric infectious disease group (GPIP) position paper on the immune debt of the COVID-19 pandemic in childhood, how can we fill the immunity gap? Infect Dis Now. (2021) 51:418–23. doi: 10.1016/j.idnow.2021.05.004, PMID: 33991720 PMC8114587

[79] Debus M Tosun J. Political ideology and vaccination willingness: implications for policy design. Pol Sci. (2021) 54:477–91. doi: 10.1007/s11077-021-09428-0, PMID: 34149102 PMC8206899

[80] Hotez PJ. Will anti-vaccine activism in the USA reverse global goals? Nat Rev Immunol. (2022) 22:525–6. doi: 10.1038/s41577-022-00770-9, PMID: 35915141 PMC9340755

[81] Donatella DP Lavizzari A. Waves in cycle: the protests against anti-contagion measures and vaccination in Covid-19 times in Italy. PARTECIPAZIONE E CONFLITTO. (2023) 15:720–40. doi: 10.1285/i20356609v15i3p720

[82] Raffini L Penalva-Verdú C. The problematic relationship between science, politics and public opinion in late modernity: the case of the anti-vax movement in Spain and Italy In: Ziya HE Giorgi A, editors. Populism and science in Europe, Palgrave Studies in European Political Sociology. Cham: Springer International Publishing (2022). 141–62.

[83] Santirocchi A Spataro P Alessi F Rossi-Arnaud C Cestari V. Trust in Science and Belief in misinformation mediate the effects of political orientation on vaccine hesitancy and intention to be vaccinated. Acta Psychol (Amst). (2023) 237:103945. doi: 10.1016/j.actpsy.2023.103945, PMID: 37210865

[84] Ward JK Alleaume C Peretti-Watel P Peretti-Watel P Seror V Cortaredona S. The French Public’s attitudes to a future COVID-19 vaccine: the politicization of a public health issue. Soc Sci Med. (2020) 265:113414. doi: 10.1016/j.socscimed.2020.113414, PMID: 33038683 PMC7537647

[85] Zehring M Domahidi E. German Corona protest mobilizers on telegram and their relations to the far right: A network and topic analysis. Social Media + Society. (2023) 9:20563051231155106. doi: 10.1177/20563051231155106

[86] Brandmayr F. Public epistemologies and intellectual interventions in contemporary Italy. Int J Politics Cult Soc. (2021) 34:47–68. doi: 10.1007/s10767-019-09346-3, PMID: 33686320 PMC7931160

[87] Troiano G Nardi A. Vaccine hesitancy in the era of COVID-19. Public Health. (2021) 194:245–51. doi: 10.1016/j.puhe.2021.02.025, PMID: 33965796 PMC7931735

[88] Amarasekara I Grant WJ. Exploring the YouTube science communication gender gap: A sentiment analysis. Public Underst Sci. (2019) 28:68–84. doi: 10.1177/0963662518786654, PMID: 29974815

[89] Levant RF McDermott RC Pryor S Barinas J. Masculinity and compliance with Centers for Disease Control and Prevention recommended health practices during the COVID-19 pandemic. Health Psychol. (2022) 41:94–103. doi: 10.1037/hea0001119, PMID: 34843265

[90] Mahalik JR Burns SM Syzdek M. Masculinity and perceived normative health behaviors as predictors of Men’s health behaviors. Soc Sci Med. (2007) 64:2201–9. doi: 10.1016/j.socscimed.2007.02.035, PMID: 17383784

[91] Doğan İ Baruh L Cemalcilar Z Kuru O Yıldırım K Çarkoğlu A. Biased perceptions against female scientists affect intentions to get vaccinated for COVID-19. Public Understanding of Sci. (2022) 31:239–51. doi: 10.1177/09636625211060472, PMID: 34847812

[92] Harteveld E Van Der Brug W Dahlberg S Kokkonen A. The gender gap in populist radical-right voting: examining the demand side in Western and Eastern Europe. Patterns of Prejudice. (2015) 49:103–34. doi: 10.1080/0031322X.2015.1024399

[93] Rydgren J. The Oxford handbook of the radical right. Oxford, UK: Oxford University Press (2018).

[94] Shahvisi Arianne. (2020). “Manspreading · LRB 30 may 2020.” London Review of Books Blog (blog) May 30, 2020. Available at: https://www.lrb.co.uk/blog/2020/may/manspreading.

[95] Oswald A Powdthavee N. Daughters and left-wing voting. Rev Econ Stat. (2010) 92:213–27. doi: 10.1162/rest.2010.11436

[96] Graff A Kapur R Walters SD. Introduction: gender and the rise of the global right. Signs J Women Cult Soc. (2019) 44:541–60. doi: 10.1086/701152

[97] Whitehead AL Perry SL. Taking America Back for god: Christian nationalism in the United States. Oxford, UK: Oxford University Press (2020).

[98] Auxier B. Anderson M. (2021). “Social media use in 2021. Pew research center.” Available at: https://www.pewresearch.org/internet/2021/04/07/social-media-use-in-2021/. Archived at https://web.archive.org/web/20230711100814/https://www.pewresearch.org/internet/2021/04/07/social-media-use-in-2021/.

