# Supplementary material for: Use of immunology in news and YouTube videos in the context of COVID-19: politicisation and information bubbles
Source: Front Public Health. 2024 Feb 7;12:1327704. doi: 10.3389/fpubh.2024.1327704 (PMC10906096; doi:10.3389/fpubh.2024.1327704)
Supplement: Supplementary file 1 [file Data_Sheet_1.docx]

SUPPLEMENTARY INFORMATION

**Table S1. Search strategy: terms used for the immunological concepts**

| **Immunological concept** | **Synonyms** | **Italian** |
| --- | --- | --- |
| Cytokine storm |  | Tempesta citochinica |
| Oxidative stress |  | Stress ossidativo |
| Immunity debt |  | Debito immunitario |
| Hygiene hypothesis |  | Ipotesi igienica |
| Herd Immunity | Population immunity | Immunità di gregge, immunità di popolazione, immunità di comunità |
| Viral interference |  | Interferenza virale |
| Antibody-dependent enhancement |  | Antibody-dependent enhancement |
| T-cell exhaustion | T-cell anergy, immunoparalysis | Anergia/esaurimento delle cellule T, esaurimento dei linfociti, immunoparalisi |
| Original antigenic sin | Immune impritning | Peccato originale antigenico, imprinting immunitario |

**Table S2: Quotes from politically-oriented videos in English with conspiratorial, right wing or libertarian views.** Immunological concepts are in bold, ideological concepts in italics.

| “I don't think you're a Moaning Minnie I think you're a Realism Rita, because let's be absolutely clear, Julia... there are thousands of small business owners... who have had to take out these massive loans and I think, Julia, the problem that we're experiencing, you and I, on this side of the argument, the reason why we have people saying ‘well you're being selfish’ and all the rest of it and just assume that we're just desperate to get into the pub or whatever, they haven't realized I think and I'm not meaning to sound patronizing in any way shape or form but *they're not realizing the massive scale of debt the businesses have to drag themselves out of because of the lockdown restrictions* over the last year.” – Darren Grimes, Talk TV (YouTube.com/watch?v=PBxyxqGcLyU) |
| --- |
| “Putting it more simply was microbiologist Professor Brett Finlay who said: ‘Because of the whole Covid lockdown kids are being **immune-deprived** and their microbes are being screwed up. So their immune systems are a bit screwed up.’ Can't get more simple than that. *These consequences were obvious to anyone with a fully functioning frontal cortex and yet those of us who raised these sort of concerns were shouted down by the Covid catastrophists* who were focused solely on one health issue to the exclusion of all others.” – Rita Panahi, Sky News Australia (YouTube.com/watch?v=ncupSokf-cQ) |
| “Basically the *government and the corrupt media have been pushing Covid fear porn* all over the country so that people will happily give away their liberties in the hopes that we won't have a massive Covid outbreak in New Zealand.” – The Right Way, New Zealand (YouTube.com/watch?v=o1b-IAUH8yk) |
| “So the *media has basically become the state-run propaganda machine* and their main message right now is destroy Joe Rogan, destroy him at all costs, how dare he have Dr Robert Malone on to talk about the potential side effects of these vaccines, how dare they, when Robert Malone was one of the founders of MRNA or RNA vaccine technology so he would know. So Joe Rogan has him on and Malone starts talking about things that the media and Dr Fauci and let's just say Klaus Schwab does not want you to know about.” – Vermont Red Pill, USA (YouTube.com/watch?v=haUkrok2yjk) |
| “We're seeing more and more a discussion of a syndrome known as **original antigenic sin** so it's you know it's a reference to the biblical concept of original sin with the insertion of that word antigenic so OAS is the acronym that you see in the medical literature and this refers to a side effect of the vaccines and it's a side effect that's been known in vaccinology long predating the Covid vaccines and what it refers to is a curious artifact that when a person is exposed to a vaccine they develop antibodies for that pathogen but also highly specific antibodies for that particular variant is the simple explanation as opposed to someone whose immune system encounters the variant in the environment and they develop a more broad antibody response. And so what happens in the original antigenic sin, the OAS scenario, is that the person then subsequently encounters a variant of the original pathogen to which the antibodies are not effective but the body continues producing the original antibodies and is not aware that they're ineffective against the variant. *In previous pandemics and epidemics there has been a consensus among scientists that you don't begin vaccination until after the main activity of the pandemic has resolved and you've got a well-known established variant that you can treat because of this OAS problem.* And so there is a lot of speculation in the literature more and more all the time that one of the reasons we're seeing negative vaccine efficacy meaning that vaccinated people now appear to be more likely to be infected with Covid than unvaccinated people. And so one of the hypotheses to explain this is that we're starting to see the product of original antigenic sin.” Right Response Ministries, USA (YouTube.com/watch?v=3RIGQkSmHk4) |
| “And then we have our 5G, before you jump into this one I'd like to just give everyone a metaphor of 5G. When we were looking at 4G it was, think of it this way, a four-lane highway and once we implemented 5G it's like having an eight-lane highway with no speed limits. That's how much more intensity we are exposed to with these freaking frequencies and Dr Chris and I happened to be in the city of Wuhan uh two years ago and that's the city where as we know Covid uh ended up really coming out of that city in China, and one of the things that we'd like you to understand is that *they were the first city in China to implement over 10,000 5G towers in their city in 2019. So we think that there's a correlation here. Dr Chris would you like to explain why 30 days later we have the coronavirus*.” TeloYouth Channel, USA (YouTube.com/watch?v=fX8gXWnEK20) |
| “We started seeing headlines like anti-lockdown protest highlights need to combat misinformation online - the minister Helen McEntee said: ‘There's a number of things I think we need to look at, firstly how do we work with social media companies to make sure that information that is incorrect is taken down.’ And therein lies the problem - who will say what is misinformation and what is fact? If the minister really wanted to crack down on misinformation perhaps she should lead by example and begin looking within her own Government ranks... the minister went on to say how the protests set out to cause disruption and harm, while those that took part in the riot will face legal sanctions and how she and every other minister condemned thuggish behaviour and the attack on the Gards. Where were they all when the Black Lives Matter people were attacking the Gards with rocks a few months ago? But don't be mistaken there's no agenda here, no, nothing to see here at all. As we know the media in Ireland allow politicians to speak practically ex cathedra with zero transparency *and then they wonder why people set up groups and channels to allow others to ask questions, to question from a different perspective*.” Dan’s Mix Tape, Ireland (YouTube.com/watch?v=jCkTgtKsaSs) |

**Table S3: Quotes from politically-oriented Italian videos classed as anti-vaccine.** Highlights in bold; English translation in italics.

| c'è gente che non c'è più, c'è gente che invece ha delle conseguenze neurologiche permanenti, c'è gente che c'ha conseguenze autoimmunitari permanenti e c'è gente che sviluppa ictus e infarti le trombosi ma questo credo ormai insomma lo sappiamo tutti. . . cercate, ecco, quei medici che hanno il coraggio in questo momento comunque di metaforicamente abbracciare, di prima comprendere il dolore che state attraversando . . . riconoscere il fatto di avere davanti un essere umano e quindi trattarlo come tale  *there are people who are gone, there are people who instead have permanent neurological consequences, there are people who have* ***permanent autoimmune consequences and there are people who develop strokes and heart attacks the thromboses*** *but this I think by now in short we all know . . . look for, here, those doctors who have the courage right now anyway to metaphorically embrace, to first understand the pain that you are going through . . . recognize the fact that you have a human being in front of you and then treat them as such .*  *(YouTube video ID AcQvehFwGb4).* |
| --- |
| Uno studio del dipartimento della Difesa americano afferma che la vaccinazione antinfluenzale aumenta del 36% l'incidenza di coinfezione proprio da coronavirus per interferenza virale. L'antinfluenzale ha scarsissima efficacia. . . . la pandemia potrebbe essere l'occasione per integrare e sostituire l'approccio basato sulla teoria dei germi con quello della teoria del terreno, che dice che ogni microbo, ogni patogeno, anche quelli che ormai sono spariti con il Covid, cambiano in relazione all'ambiente in cui viviamo. E quindi il sistema immunitario potrebbe essere potenziato con una medicina biologica integrata, con un'alimentazione vitalizzante sana, l'integrazione di minerali, vitamine, probiotici, OMEGA tre, azioni antinfiammatoria e COSI qualsiasi patogeno potrebbe essere appunto superato dal nostro sistema immunitario.  *A U.S. Department of Defense study states that* ***flu vaccination increases the incidence of co-infection by 36 % just from coronavirus due to viral interference****. The flu shot has very little efficacy. . . . the pandemic could be an opportunity to supplement and* ***replace the germ theory approach with the terrain theory approach****, which says that every microbe, every pathogen, even those that are now gone with Covid, change in relation to the environment in which we live. And so* ***the immune system could be enhanced with integrated biological medicine, with vitalizing healthy nutrition, supplementation of minerals, vitamins, probiotics, Omega-3****, anti-inflammatory actions, and that way any pathogen could be precisely overcome by our immune system.*  (YouTube video ID J8r3OECBIoc) |
| dovrebbe far suonare qualche campanello d'allarme proprio perché nella mia memoria non esiste un vaccino la cui efficacia e diventa negativa . . . esistono dei grafici che dimostrano in maniera chiara che la linea dei contagiati e la protezione offerta cala e va al di sotto dello zero in alcuni che in alcuni studi anche molto al di sotto dello zero . . . per due anni molta gente ha sofferto perché è stata è stato riservato un trattamento fortemente disumano con dei connotati vi disprezzo civile quasi qualcuno che avesse qualche dubbio a effettuare questo tipo di terapia fosse un terrorista comunque un terrario artista una persona sicuramente ignorante contrapposte invece al virtuoso cittadino che fa quello che la scienza dice . . . tutto questo ha portato comunque a una enorme disumanizzazione dei processi dei processi umani direi è noto colleghi che sono arrabbiati noto le persone che sono arrabbiate c'è un grande malcontento comunque da entrambe le parti si spera che le persone invece mantengano una tranquillità perchè in questo momento la rabbia o la confusione non non fanno altro che offuscare il pensiero  *. . . should ring some alarm bells precisely because in my memory there is no vaccine whose effectiveness and become negative . . . there are graphs that clearly show that the line of the infected and* ***the protection offered drops and goes below zero*** *in some that in some studies even far below zero . . . for two years a lot of people have suffered because they have been given highly inhumane treatment with connotations vi civil contempt almost as if someone who had any doubts about carrying out this kind of therapy was a terrorist . . . contrasted instead with the virtuous citizen who does what science says . . all of this has led anyway to a tremendous* ***dehumanization of the processes of human processes*** *I would say it is known colleagues who are angry I notice people who are angry there is a great discontent however on both sides hopefully people instead maintain a calmness because right now anger or confusion only clouds thinking*  (YouTube video ID bzEBNzyl8UA) |
| Precedentemente i vaccini, i cosiddetti mRNA, le terapie mRNA, per esempio, venivano riservate come farmaco orfano, cioè un farmaco che viene utilizzato in maniera compassionevole per patologie che non hanno alcun tipo di terapia. Eh? Quindi erano estremamente limitati sia nel tempo sia che nell'applicazione, prima invece di fare un. Un utilizzo su larga scala su un miliardi di persone, come è stato fatto? . . . Perché può succedere in medicina succede molto spesso che alcune problematiche di alcuni farmaci si sono identificate a quattro o 5 o anche a 10 anni di distanza. Quindi l'idea di dire io l'ho dato a fossero anche tre miliardi di persone. Il fatto che lo abbia dato così tante persone non significa che non sia sperimentale  *Previously vaccines, so-called mRNA, mRNA therapies, for example, were reserved as an orphan drug, that is, a drug that is used compassionately for diseases that have no therapy. Huh? So they were extremely limited both in time and application, before instead of making a. Large-scale use on a billion people, how was it done? . . . Because* ***it can happen in medicine it happens very often that some issues of some drugs have been identified four or five or even 10 years later****. So the idea of saying I gave it to were even three billion people. The fact that I gave it to so many people does not mean that it is not experimental*  (YouTube video ID zEIe64A2Fx8) |
| esistono già ampie evidenze che le vaccinazioni antinfluenzali porta. Colleghi. Casi Covid in più per interferenza virale, come se non si sapesse che il coronavirus ha un cofattore, quindi indicatori di altre criticità ambientali, l'inquinamento, anzitutto anche quello elettromagnetico, con i suoi effetti nocivi. . . . nel pieno ossequio di questo scientismo della Santa Inquisizione da salotto televisivo. Si possono sacrificare il personale militare, i poliziotti, il personale medico, i nostri figli, i nostri anziani. Alle sperimentazioni di un vaccino che sappiamo e lo sanno anche i sassi, non servirà a nulla per la mutevolezza del virus. . . . Sottomettendosi ai giochi nel geopolitici tra Stati Uniti e Cina, lasciando che questo paese divenisse territorio di nuove guerre fatte certamente con i virus e svendendo tutte le nostre informazioni, anche quelle più private e intime, al miglior offerente.  *There is already ample evidence that flu vaccination leads. Colleagues.* ***Extra Covid cases for viral interference****, as if you didn't know that coronavirus has a cofactor, then indicators of other critical environmental issues, pollution, first and foremost also electromagnetic pollution, with its harmful effects. . . . in full deference to this scientism of the Holy Inquisition from TV living rooms. Military personnel, policemen, medical personnel, our children, our elderly can be sacrificed. To the trials of* ***a vaccine that we know and even the stones know, will be of no use*** *because of the mutability of the virus . . . . Submitting to the* ***games in the geopolitical between the United States and China****, letting this country become the territory of new wars certainly made with viruses and selling off all our information, even the most private and intimate, to the highest bidder.*  (YouTube video ID Ev1PwxHtEbg) |
